# Effective inhibitors of the essential kinase PknB and their potential as anti-mycobacterial agents

**DOI:** 10.1016/j.tube.2011.03.005

**Published:** 2011-07

**Authors:** Kathryn E.A. Lougheed, Simon A. Osborne, Barbara Saxty, David Whalley, Tim Chapman, Nathalie Bouloc, Jasveen Chugh, Timothy J. Nott, Dony Patel, Vicky L. Spivey, Catherine A. Kettleborough, Justin S. Bryans, Debra L. Taylor, Stephen J. Smerdon, Roger S. Buxton

**Affiliations:** a Division of Mycobacterial Research, MRC National Institute for Medical Research, The Ridgeway, Mill Hill, London, NW7 1AA, United Kingdom; b Centre for Therapeutics Discovery, MRC Technology, 1-3 Burtonhole Lane, Mill Hill, London, NW7 1AD, United Kingdom; c Division of Molecular Structure, MRC National Institute for Medical Research, The Ridgeway, Mill Hill, London, NW7 1AA, United Kingdom

**Keywords:** *Mycobacterium tuberculosis*, Kinases, PknB, Inhibitors

## Abstract

PknB is an essential serine/threonine kinase of *Mycobacterium tuberculosis* with possible roles in a number of signalling pathways involved in cell division and metabolism. We screened a library of >50,000 compounds for inhibitors of the *in vitro* phosphorylation of GarA (Rv1827) by PknB and identified a number of inhibitors. A program of synthetic medicinal chemistry was subsequently conducted around one class of inhibitors and was successful in generating ATP competitive inhibitors with potency in the nanomolar range. Compounds in this class showed cross-reactivity with the related *M*. *tuberculosis* kinase, PknF, but not with PknG in an *in vitro* autophosphorylation assay. These synthesised inhibitors were able to prevent the growth of *M. tuberculosis* in an Alamar blue assay and in an intracellular model of infection, but only in the micromolar range. We attempted to determine if cell wall permeability was an explanation for the discrepancy between the potent *in vitro* compared with relatively poor *in vivo* activity, but found no evidence that the activity of the inhibitors could be improved by weakening the cell wall. Despite a number of drug discovery efforts attempting to develop inhibitors against PknB, it is yet to be reported that any such inhibitors prevent mycobacterial growth at submicromolar concentrations.

## 1 Introduction

*Mycobacterium tuberculosis* remains one of the world’s most devastating pathogens, with more than 13 million people suffering from an active tuberculosis infection and 1.8 million resulting deaths in 2008 alone.^1^ The emergence of multi-drug and extensively drug resistant strains has highlighted the need for new drugs to treat tuberculosis. Recent studies have focused on finding new pathways vulnerable to inhibition by small molecules and previously unexploited by drug discovery efforts. The inhibition of signalling pathways both in *M. tuberculosis* and the host may yield new classes of drug targets and a large amount of recent work has focused on developing this further.

Target based drug discovery, in which there is *in vitro* high throughput screening of a large number of small molecules against a validated target, has been used on a number of occasions to search for new anti-tuberculosis agents. We sought to find inhibitors of an essential *M. tuberculosis* serine/threonine protein kinase, PknB. Kinases are attractive as drug targets due to the range of crucial cellular processes in which they are involved. There has been much interest in developing ATP competitive kinase inhibitors for the treatment of cancer, a hallmark of which is often aberrant kinase activity. A large number of small molecule kinase inhibitors have been developed as potential anti-cancer drugs and there is a huge amount of interest in developing kinase inhibitors to treat a range of conditions.^2^ Kinase-focused libraries of small molecule inhibitors have been built-up as a result of these studies and a large amount of knowledge has been gained on the action of kinase inhibitors. The early success stories from the development of eukaryotic kinase inhibitors suggested that similar drugs could be developed to treat bacterial infections. The *M. tuberculosis* serine/threonine protein kinases (STPKs) are attractive targets partly because of the inferred importance of serine/threonine phosphorylation in *M. tuberculosis: M. tuberculosis* is unique within the bacterial world in having a much higher number of STPKs compared to the more common two-component signalling systems.^3–6^ *Mycobacterium leprae*, during extensive reductive evolution, has lost all but four of these kinases: PknA, PknB, PknG and PknL.^7^ PknA and PknB are essential in *M. tuberculosis,*^8–10^ while PknG has been reported to be involved in survival in the macrophage, either as a result of a role in glutamate metabolism^11^ or via modulation of host cell trafficking pathways.^12^

Previous studies have focused on the potential of PknB and PknG as drug targets in *M. tuberculosis*, although the majority have reported significantly less impressive activity against whole cells than against the purified protein *in vitro*.^8,12–14^ Despite this, there can be no doubt, from a number of lines of evidence, that PknB carries out function(s) essential to *M. tuberculosis*, and possesses a number of properties that suggest it is suitable for development of novel drugs: a crystal structure has also been determined for PknB^15,16^ and the kinase domain exhibits less than 30% similarity with eukaryotic kinases, which has positive implications for developing a drug specific for the bacterial kinases and not those of the host. The *pknB* gene (Rv0014c) is part of an operon highly conserved among the actinomycetes and also encoding *pknA*, the other essential kinase; *pstP*, a phosphoserine/threonine phosphatase; *rodA*, a cell division protein; *pbpA*; a protein involved in peptidoglycan biosynthesis; and two genes (*fhaB* and *fhaA*) coding for forkhead-associated proteins. Over-expression or depletion of this kinase results in gross changes in cell morphology, likely a result of the role of PknB in the phosphorylation-mediated regulation of a number of proteins involved in cell wall biosynthesis.^10,17,18^ In addition, both PknB and PknG are known to phosphorylate a protein involved in the regulation of glutamate metabolism,^19–21^ suggesting that an inhibitor of one or both of these kinases could also impact on the ability of *M. tuberculosis* to adequately regulate its central metabolic processes. Targeting of these bacterial kinases would therefore be a way of inhibiting evolutionarily-conserved steps in central metabolic processes.

We screened for small molecule inhibitors of PknB and, as a result of a medicinal chemistry program (manuscript in preparation), our lead compounds were able to inhibit PknB activity *in vitro* in the nanomolar range. However, the potency of our compounds against whole cells in culture or in a macrophage model of infection was two orders of magnitude lower than expected from the *in vitro* potency. An often suggested explanation for low anti-tuberculosis activity is the problem of cell wall permeability. Since the *M. tuberculosis* cell wall presents an extremely hydrophobic barrier which can impede the entry of drugs into the cell, we sought to determine if cell wall permeability might explain the difficulties in improving the potency of our PknB inhibitors. In addition, we investigated the role of efflux pumps, protein binding in the assay media and inhibitor specificity as alternative explanations.

## 2 Materials and methods

### 2.1 Compounds

Compounds for the high throughput screening included the MRCT compound collection comprising 45,000 diverse templates from commercially available collections, as well as 6400 kinase-focused templates (Biofocus DPI, Cambridge, UK) selected on the basis of bio-informatics provided by the crystal structure of PknB and other serine/threonine kinases. Focused libraries, based around compounds identified from the initial screen, were subsequently generated by synthetic medicinal chemistry.

### 2.2 Protein expression and purification

GarA was expressed in *Escherichia coli* and purified as described.^22^ The 279 residue kinase domain of PknB and the 292 residue kinase domain of PknF were expressed as 3C-protease cleavable GST-fusions in *E. coli* Rosetta 2 (DE3) pLysS cells. The proteins were purified with glutathione Sepharose 4B resin (GE Healthcare) and the GST-tag cleaved from the protein prior to elution using 3C protease (GE Healthcare).

### 2.3 High throughput protein kinase assays

A non-radioactive PknB kinase assay was developed to follow ATP depletion during the PknB-catalyzed phosphorylation of GarA (Rv1827). The Promega ‘Kinase Glo’ assay kit was used to measure levels of phosphorylation via detection of the remaining ATP after the completion of the kinase reaction. Briefly, luciferase and beetle luciferin were introduced to the reaction mix and, in the presence of Mg^2+^, ATP and oxygen, the oxygenation of luciferin generates one photon of light per turnover. PknB activity is inversely proportional to the intensity of the luminescence signal. Using this assay format the K_m_ of ATP was determined to be 1.5 μM and ATP was used at this concentration for screening.

The following reaction mix was prepared: 0.15 μM PknB and 5 μM Rv1827 in reaction buffer (50 mM Tris–HCl, 150 mM NaCl, 1 mM DTT and 0.01% Triton X-100). The assay was carried out in 22 μl volumes in 384-well white plates (Matrix, 4316). A Beckman Biomek Fx liquid handling robot was used to dispense premixed reaction mix into the wells and the 2 μl of the compound/DMSO added to a final concentration of 10 μM/1% DMSO. The plates were incubated for 30 min and the reaction initiated with the addition of 1 mM MnCl_2_/1.5 μM ATP using the Perkin Elmer Flexdrop non-contact liquid dispenser. The reaction was stopped after 20 min with the addition of 22 μl Kinase Glo reagent and luminescence read with a BMG Polarstar reader. Control wells with and without PknB were included on every plate in columns 1, 2 and 23, 24 and were used to calculate Z′ values.^23^ Data from any plates with Z′<0.5 were rejected and were repeated.

### 2.4 Strains and growth conditions

*M. tuberculosis* H37Rv, *Mycobacterium smegmatis* mc^2^155, *M. smegmatis* Δ*fbpA* (gift from C. Thompson), and *M. tuberculosis* Δ*ompA* (gift from P. Draper) cultures were grown at 37 °C in Dubos broth supplemented with 0.05% (vol/vol) Tween 80, 0.2% (vol/vol) glycerol, and 4% (vol/vol) Dubos medium albumin (Becton Dickinson). *M. smegmatis* strains were grown either in 5 ml volumes in 20 ml universals or in 50 ml volumes in 250 ml flasks, incubated in an orbital shaker at 150 rpm at 37 °C. *M. tuberculosis* was grown either in 100 ml volumes in a Bellco roll-in incubator (2 rpm) or in 10 ml volumes in static universals. Kanamycin was used at a final concentration of 25 μg ml^−1^. *M. tuberculosis* was grown on 7H11 supplemented with 10% OADC enrichment (Middlebrook) and 0.5% (vol/vol) glycerol.

### 2.5 *M. tuberculosis* minimum inhibitory concentration (MIC) determination

MICs were determined for *M. tuberculosis* using a 96-well plate method with Alamar blue, as described previously.^24,25^ A modified version of the assay used *M. smegmatis* with a 3 day incubation. The cell wall weakening and efflux pump inhibition assays were modifications of the Alamar blue assay, as described in the results. Z′ values were used as a measure of assay quality, using the following formula: Z′ = 1–3 × SSD/R, where SSD is the sum of the Standard Deviation of the negative controls and Standard Deviation of the positive controls, and R is the mean of the maximum signal control minus the mean of the negative signal control.^23^

### 2.6 Intracellular activity of PknB inhibitors in a macrophage infection by *M. tuberculosis*

Intracellular inhibition was tested in murine bone marrow-derived macrophages at a range of concentrations as previously described.^25^ Briefly, monocytes were isolated from Balb/c bone marrow and differentiated with L-cell supernatant for 5–7 days. The macrophages were infected with *M. tuberculosis* with an MOI of 0.5 bacteria: 1 macrophage for 4 h, and the infection incubated in the presence of various concentrations of the inhibitors for 5 days. At this point, the macrophages were lysed with dH_2_0 + 0.05% Tween 80 and plated for CFU enumeration. Prior to the infection, the compounds were tested to rule out any effect of toxicity against the macrophages. Alamar blue was used to determine macrophage viability after incubation with the inhibitors (not shown).

### 2.7 Kinase autophosphorylation assay

*In vitro* autophosphorylation of PknA, PknB, PknG and PknF (1 μg) was carried out in 20 μl reaction mixture containing 2.5 μl buffer P (25 mM Tris–HCl, pH 7.0, 1 mM dithiothreitol, 5 mM MgCl_2_, 1 mM EDTA). The reaction was initiated with the additional of 0.08 μCi [γ-^32^P] ATP and incubated at 37 °C for 30 min. The reaction was terminated with the addition of SDS-PAGE sample buffer and heating the mixture at 100 °C for 5 min. The reaction mixtures were analyzed by SDS-PAGE. The gels were soaked in 20% tricholoroacetic acid for 10 min at 90 °C and dried. Radioactive proteins were visualized by autoradiography and quantified by Phosphorimage analysis using a STORM 840 Optical Scanner System and ImageQuant (version 5.2, Molecular Dynamics, Sunnyvale, CA). Images were adjusted for contrast and brightness using Adobe Photoshop.

### 2.8 Radioactive competition assay

Reactions were set up in a volume of 17 μl kinase buffer (50 mM Tris–HCl pH7.2, 1 mM DTT, 0.01% TX-100) containing 100 nM purified PknB and 3 μM purified Rv1827 and increasing amounts of each inhibitor diluted in neat DMSO (from 0.03 μM to 100 μM) in polypropylene U-bottomed 96 well plates. The reactions were started by the addition of 5 μl of solutions containing MnCl_2_ and ATP at K_m_ or 50xK_m_, to achieve final concentrations of 1 mM MnCl2 with 1.5 μM ATP and 0.1 μCi ^33^P or 75 μM ATP and 1 μCi ^33^P.

After incubating for 70 min at room temperature, reactions were terminated by the addition of 20 μl 50% orthophosphoric acid. Reactions were then transferred to a 96 well glass filter capture plate (GF/C) and unbound ^33^P was washed away with 200 μl 1.5% orthophosphoric acid, using a Tomtec Harvester 9600. After drying overnight at room temperature, 25 μl scintillant was added to each well and quantification of Rv1827 phosphorylation achieved by detecting incorporated ^33^P with the PE Topcount.

### 2.9 Kinase profiling against a panel of mammalian kinases

This was carried out using a ^33^P-ATP filter-binding assay^26^ on a panel of 70 mammalian kinases at the National Centre for Protein Kinase Profiling in the MRC Protein Phosphorylation Unit at the University of Dundee.

### 2.10 Docking studies with PknB inhibitors

The mitoxantrone bound crystal structure (PDB:2FUM) was selected for docking studies owing to its greater suitability in terms of ligand-induced binding site architecture as compared to the ATP bound structure (PDB:1MRU). Protein preparation, grid generation and docking (GlideSP) were all carried out using the Schrodinger molecular modelling suite (Schrodinger LLC: New York. http://www.schrodinger.com).

## 3 Results

### 3.1 High throughput screening for PknB inhibitors

PknB has been previously validated as a suitable target for drug discovery and we sought to identify small molecule inhibitors targeted against this kinase. A number of potential substrates for PknB were tested, with GarA (Rv1827) being chosen as the most strongly phosphorylated protein in our assay. GarA has been described as an *in vivo* target for both PknB and PknG^19,20^ and interaction with this target is known in some detail.^22,27^ A non-radioactive PknB kinase assay was developed to follow ATP depletion during the PknB-catalyzed phosphorylation of GarA. Substrate K_m_s were determined for both ATP (1.5 μM) and Rv1827 (>20 μM). The K_m_ ATP concentrations and an excess of peptide (5 μM) were selected to achieve a robust assay window >0.5 within the linear phase of the reaction, maximizing sensitivity to ATP competitive inhibitors.

In total ∼54,000 compounds were screened against PknB. The compound collection was sourced by MRCT from commercial suppliers. Drug-like filters to assess both physicochemical properties and known toxicophores were applied to the selection process. The MRCT library comprised ∼44,000 diverse compound collection and ∼9000 kinase-focused collection (targeting ATP binding site). In addition, ∼1000 natural products (Phytoquest) were screened. The average Z′ was 0.76 and a confirmed hit rate of 0.14% was observed. In total, 76 compounds confirmed IC_50_ in the range 1–200 μM.

Limitations of the Kinase glo assay format did not permit detailed enzyme kinetics to be performed. However using a radioactive assay, a number of compounds were assessed for ATP competitiveness by measuring IC_50_ at a high (75 μM) and a low (1.5 μM, Km) ATP concentration. From the Cheng–Prussoff equation^28^ we would predict that for an ATP competitive inhibitor, a 25-fold shift in IC_50_ would be seen between ATP at K_m_ (1.5 μM) and 50XK_m_ (75 μM). Five compounds from the series, including the original screen hit, with a range of potencies 50–1000 nM in the primary assay were selected. Fold shifts in the range 15.5–32 were observed for all compounds, consistent with an ATP competitive mechanism. It was expected that, due to the higher ATP concentration in the cells compared to the *in vitro* assay, a shift in MIC compared to IC_50_ enzyme activity would be observed. However, a consistent shift was not observed.

**Figure 1:**
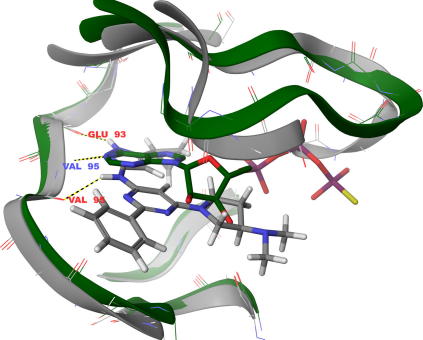
Docking of MRCT67127 into PknB active site. Green ribbon represents PknB (Code: 1MRU) with ATP co-crystallised (shown as green structure). Grey ribbon represents PknB (Code: 2FUM) with mitoxantrone co-crystallised. The mitoxantrone structure was removed and docking studies conducted with this PknB conformation: owing to the flat nature of the docked compounds (making them similar to mitoxantrone), this was considered a better way to obtain reliable results. MRT67127 is shown (grey structure) docked into PknB.

Docking studies (Figure 1) indicate that the compounds should be ATP competitive. They bind to the hinge region via two H-bonds between the ligand core and the peptide backbone of Val95 along with an additional H-bond via the ligand pyrazole NH to the peptide carbonyl of Glu93. Numerous Van Der Waals contacts contribute to binding, most notably between the ligand cyclopropyl group in the hydrophobic pocket towards the Met92 gatekeeper and also via extensive contact along the glycine rich loop at the entrance to the binding site.

### 3.2 Anti-tuberculosis activity of lead compounds

Compounds that showed potent activity against PknB *in vitro*, no cytotoxicity and good selectivity against a panel of mammalian kinases, were tested in an *M. tuberculosis* Alamar blue MIC assay. Briefly, 2-fold dilutions of the inhibitors were incubated at 37 °C with approximately 1 × 10^5^ cells per well in 96-well microplates for 7 days. Untreated *M. tuberculosis* and media only controls were included on every plate, and the control drugs isoniazid, streptomycin and ethambutol were tested in parallel with each batch of PknB inhibitors to ensure that the assay was reproducible. The Alamar blue reagent was added on the final day of the incubation, and the fluorescence measured at the completion of the experiment. A 90% inhibition of fluorescence compared to the untreated control was chosen as the cutoff for the determination of inhibitory concentrations. The statistical parameter, Z′-factor, was used as a measure of assay quality, with a score greater than 0.75 taken as an indication of good data.

**Figure 2:**
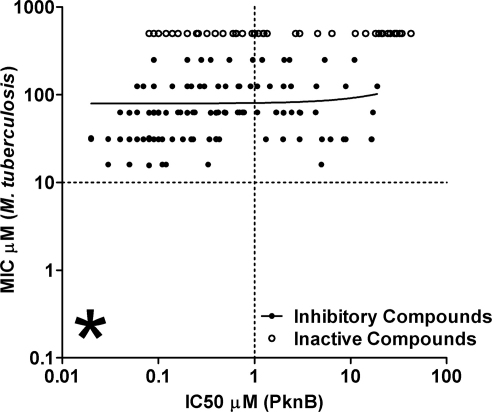
Compounds inactive against *M. tuberculosis* had a range of IC_50_s against the purified protein. A similar spread was observed for the compounds able to inhibit *M. tuberculosis*. Starred section indicated the area of the graph where compounds highly active against both the purified protein and whole cells would appear.

MICs were determined for more than 100 compounds, with a range of MICs being observed varying between inactive at 250 μM to inhibitory at 8–16 μM. The compounds were also tested against *M. smegmatis* (data not shown) with a generally good agreement between the two assays. This confirms previously published experiments (e.g.^29^) which have suggested that testing compounds against *M. smegmatis* is a good model for TB drug discovery. There was a poor correlation between IC_50_ against the purified recombinant protein and anti-*M. tuberculosis* MIC, with the majority of compounds proving to be inactive against the whole cells. The lack of correlation between protein and whole cell activity is illustrated in Figure 2. Despite extensive rounds of chemistry, unfortunately it was not possible to reduce the *M. tuberculosis* MICs below the 10 μM level.

### 3.3 Intracellular activity of PknB inhibitors against *M. tuberculosis* in a bone marrow derived macrophage model of infection

A subset of compounds was chosen for further investigation. The key criteria in this selection included reasonable activity against *M. tuberculosis in vitro*, a low MIC to IC_50_ ratio as a possible indication of good cell wall permeability, good selectivity against a panel of human kinases and good physicochemical parameters, including moderate logD and low hydrogen bond donor count, both of which are requirements for good membrane permeability. The intracellular activity of these compounds against *M. tuberculosis* in a bone marrow derived macrophage model of infection was tested.

**Figure 3:**
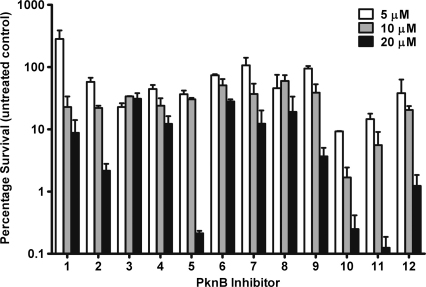
Intracellular activity of PknB inhibitors in a bone marrow derived macrophage model. Macrophages were infected with an MOI of 0.5:1 and incubated in the presence of a dilution series of the inhibitors. After 5 days, the macrophages were lysed and the intracellular bacteria enumerated by CFU platings. Data is expressed as percentage survival compared to the untreated control. Inhibitor structures and corresponding *in vitro* activity against purified protein are shown in Table 1.

The compounds showed a dose dependant inhibition of intracellular *M. tuberculosis*, although a number of the compounds were not able to inhibit 99% growth compared to the untreated control (chosen as the cutoff for determining intracellular inhibition). Figure 3 shows the inhibition of intracellular growth by the 12 PknB inhibitors chosen for further investigation. At the lowest concentration tested, 2.5 μM, none of the compounds were able to inhibit growth by more than 99% of the control. A number of the compounds were active at the higher concentrations of 10 and 20 μM. While concentrations up to 100 μM were tested, these are not shown due to a number of the compounds proving to have toxic effects against the macrophages, possibly due to inhibition of host kinases.

A summary of the data is shown in Table 1. Little correlation between intracellular activity and whole cell inhibition was found.

### 3.4 Role of cell wall permeability and efflux on inhibitor MIC

Poor anti-tuberculosis activity by compounds that are potent in the *in vitro* protein assay is often attributed to impermeability of the *M. tuberculosis* cell wall. *M. tuberculosis* has a thick and ‘waxy’ cell wall, which limits the ability of compounds to penetrate and reach the cytoplasm.^30^ Much of the impermeability stems from the unique mycolic acid layer. While little is known about the specific properties of small molecules that enable them to diffuse across the *M. tuberculosis* cell wall, it is possible to modify certain functional groups of the compounds in ways that will hopefully improve entry into the cytoplasm. For example, modifications were made to our more active compounds in an attempt to vary the compounds’ LogP values and increase their hydrophobicity (to be discussed in a separate medicinal chemistry paper). In one case, it was possible to improve the MIC/IC_50_ ratio using this approach. However, the resulting activity of the compounds against *M. tuberculosis* was still poor. Before embarking on further chemistry with the hope of improving cell penetration, we attempted to determine whether cell wall permeability truly was a major contribution to low MIC values through the use of several cell wall-weakened models with the subset of inhibitors shown in Figure 3.

A number of chemicals and antibiotics were tested, including Triton X-100, glycine, lysozyme, isoniazid and ethambutol. There have been previous reports of sub-lethal concentrations of antibiotics targeted to the cell wall being able to enhance permeability of certain compounds.^31,32^ In particular, ethambutol has been demonstrated to enhance the activity of rifampicin, streptomycin and isoniazid against drug resistant strains.^33^ In our experiments, the cell wall weakening agent was used at 0.5 MIC in conjunction with the PknB inhibitors to determine whether the inhibitors were better able to inhibit *M. tuberculosis* in the presence of a weakened cell wall. As a control, we demonstrated that the MIC of rifampicin was reduced by 4-fold in the presence of sub-inhibitory concentrations of ethambutol, confirming published data that reported that accumulation of and sensitivity to rifampicin is enhanced by ethambutol.^34^ We tested a number of compounds, including the subset of 12 active compounds and a number that were previously inactive against *M. tuberculosis*. Several compounds were able to inhibit *M. tuberculosis* in the presence of ethambutol when they had been previously inactive. This implied that cell wall permeability was, indeed, a problem for some structural families. However, the MICs of the most efficacious compounds were not improved by ethambutol treatment.

In addition to the ethambutol cell-wall weakened model, we wanted to use a more defined model. An *M. smegmatis fbpA* mutant has been shown to possess increased sensitivity to a number of antibiotics due to alterations in its cell wall.^35^ FbpA is involved in mycolic acid biosynthesis and the mutant has a loss of cell wall hydrophobicity compared with the wild type. We tested the sensitivity of this mutant to the PknB inhibitors and, in agreement with the ethambutol data, there was no improvement in inhibition with a weakened cell wall. In comparison, vancomycin MIC was improved by 16-fold and erythromycin by 8-fold. This effect was specific to certain antibiotics as the MICs and streptomycin and ethambutol were not improved in the assay.

After testing the role of the mycolic acid layer in drug permeability, we sought also to investigate other possible paths of entry into the cell. Antibiotics can either diffuse across the cell wall, a possibility investigated in the experiments detailed above, or alternatively may enter through hydrophilic channels. Porins in the mycobacterial outer membrane are required for the diffusion of hydrophilic solutes and are the route of entry for small and hydrophilic antibiotics such as isoniazid and ethambutol.^36^ It has been demonstrated that an *mspA* porin deletion mutant of *M. smegmatis* has a multi-drug resistant phenotype to antibiotics including ampicillin.^37^ Although *M. tuberculosis* does not encode Msp-like proteins, expression of *M. smegmatis* MspA in *M. tuberculosis* enhances antibiotic sensitivity^38^ and an *M. tuberculosis* outer membrane channel-forming protein has been demonstrated to restore antibiotic sensitivity to a *M. smegmatis mspA* mutant.^39^ One of the best understood porins in *M. tuberculosis* is OmpA, that has been linked to the diffusion of pyrazinamide across the cell wall.^40^ *M. tuberculosis ompA* mutants have not been reported to have an antibiotic resistant phenotype similar to *M. smegmatis mspA.* Therefore, we tested the sensitivity of the mutant to a number of antibiotics compared to the wild type strain. Unlike *M. smegmatis mspA, M. tuberculosis ompA* did not have increased sensitivity to the small, hydrophilic antibiotics tested. However, it did show a slightly increased resistance to the large antibiotics vancomycin and erythromycin. This result was also observed for *M. smegmatis mspA*, despite both of these antibiotics being too large to enter the cell via porins. It is likely that alterations in the cell wall have a knock-on effect on entry of large antibiotics into the cell. This experiment does not provide any evidence that small antibiotics enter *M. tuberculosis* via OmpA channels, but we cannot rule out that OmpA is involved in the uptake of other antibiotics not tested. We also tested the *ompA* mutant with the PknB inhibitors and observed that the majority of them were equally active against the wild type and mutant.

The activity of efflux pump inhibitors has been shown to play a role in the acquired drug resistance of *M. tuberculosis* for a number of antibiotics,^41–44^ due to the upregulation of efflux pumps in drug resistant strains. It has been suggested that the use of efflux pump inhibitors in conjunction with anti-tuberculosis therapy could be used to reverse the efflux pump-mediated drug resistance in some infections.^45^ There is also some evidence that efflux plays a role in the intrinsic drug resistance of mycobacteria.^46–48^ Therefore, we tested whether the addition of sub-inhibitory concentrations of the efflux pump inhibitors reserpine and verapamil to our assay would improve the PknB inhibitor MICS. No change was observed in MIC at either 20 or 40 μg/ml of either efflux pump inhibitor, while the MIC of the ethidium bromide control was decreased by 4-fold. This is perhaps not surprising due to the observation that the effect of efflux inhibitors is most dramatic when there is overexpression or upregulation of efflux pumps, rather than against wild type levels.^34,45^

### 3.5 Specificity of PknB inhibitors

Attempts to select resistant mutants of the 12 most potent PknB inhibitors were unsuccessful, possibly due to either the high MICs of the compounds, or non-specific activity of the inhibitors. We plated 5 × 10^9^ *M. tuberculosis* directly on to media containing 2, 4 and 8×MIC inhibitors as well as pre-growing the cells on 0.5 and 1×MIC before replating on the above concentrations. Neither method generated any resistant colonies. This, combined with the lack of correlation between MIC and activity in the *in vitro* protein assay led us to consider whether the inhibitors are truly targeting PknB. It is possible that some of the anti-tuberculosis activity of the inhibitors is due to off-target activity, meaning that further PknB-focused chemistry based around these structural families is unlikely to lead to an improvement in MIC. Alternatively, despite good selectivity over mammalian kinases, the inhibitors could be binding to a number of the other more closely related *M. tuberculosis* kinases, and it is possible that the inhibitor is being sequestered away from PknB by its activity against any of the eight non-essential kinases in *M. tuberculosis*.

We used an *in vitro* radioactivity based screen to measure the ability of the compounds to inhibit autophosphorylation of some of the other *M. tuberculosis* kinases. PknA, the other essential kinase, was tested but, due to high levels of stable phosphorylation of this kinase following purification, it was not sufficiently active in the assay. Due to the essential nature of PknA, we were not concerned about possible cross-reactivity of the PknB inhibitors with this kinase. In fact, this could be advantageous for the development of a TB drug. We also tested the ability of the inhibitors to inhibit PknF and PknG autophosphorylation. PknG is a soluble kinase while PknF is membrane-associated like PknB; both are non-essential.

**Figure 4:**
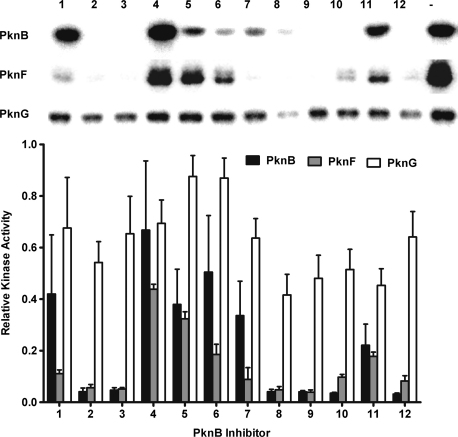
*In vitro* activity of the PknB inhibitors against PknF and PknG. The inhibitors were tested at 1 μM (not shown) and 10 μM for their ability to inhibit PknB, PknF and PknG in an autophosphorylation assay. There was good agreement between the anti-PknB activity in this assay and the non-radioactive assay used in the high throughput screen. The graph was created using the averaged band intensities from three independent autoradiographs.

It was observed that there is a high degree of cross-reactivity between the PknB inhibitors and PknF, the other membrane-associated kinase tested. However, PknG was less strongly inhibited at the concentrations tested (Figure 4). A possible explanation for this is the 39.5% sequence identity shared between PknB and PknF compared to only 27.3% between PknB and PknG.

## 4 Discussion

PknB has been shown to be an essential protein in *M. tuberculosis,*^8^ and is proposed to be involved in a number of vital pathways. Initially, it was implicated as having a role in cell division due to its chromosomal location within an operon containing genes known to be important for these processes. Depletion or overexpression of PknB results in gross changes in cell morphology, and some of its identified substrates are known to be important for cell wall and division-related functions.^10^ The cell wall is known to be a strong target for anti-tuberculosis drugs, due to its importance for the survival of the pathogen and its uniqueness. PknB is most strongly expressed during exponential growth^10^ and upon infection of a THP-1 macrophage cell line,^49^ suggesting that the kinase is involved in general growth and important during an infection. More recently, it has been seen that one of PknB’s substrates, GarA, has a role in intermediary metabolic processes. GarA negatively regulates two proteins involved in metabolism, 2-hydroxy-3-oxoadipate synthase and glutamate dehydrogenase and is itself negatively regulated via phosphorylation by PknB.^20–22^ Interestingly, GarA is also known to be phosphorylated *in vivo* by PknG on a neighbouring threonine to that phosphorylated by PknB.^20^

The known functions of PknB detailed above do suggest that this kinase would be a strong candidate for an anti-tuberculosis drug. We found a number of inhibitors able to inhibit PknB *in vitro* with IC_50_s in the 20 nM range. The MICs of these inhibitors against *M. tuberculosis* were found to be in the 16 μM to inactive range, despite several rounds of compound optimization. Some shift in MIC compared to IC_50_ was expected due to higher intracellular concentrations of ATP leading to competition. However, a consistent shift was not observed across the series, suggesting additional mechanisms are involved in the poor activity against whole cells. It should also be noted that the intracellular concentration of ATP in *M. tuberculosis* is in the micromolar range^50^ (A. Chang, unpublished data), which is significantly lower than that the accepted millimolar concentrations for mammalian cells. The poor MIC to IC_50_ ratios appear to be a similar result to the published data of other groups who have also attempted to develop PknB or PknG inhibitors.^8,12–14^ This poor activity was evident even in an intracellular assay.

We investigated the role of cell wall permeability, either diffusion through the hydrophobic cell wall or diffusion through hydrophilic channels, and found no evidence that this was a limiting step in the action of the inhibitors. We also investigated the role of efflux and observed that inhibitor activity was not improved in an efflux pump inhibited model.

A possibility is that the more potent inhibitors, which were chosen for compound optimization, were not targeting PknB in the cells. A similar problem was described by Payne et al.^51^ in their review of GlaxoSmithKline’s 7 years of antibacterial high throughput screen (HTS) research. They attributed the antibacterial activity of some of their lead compounds to non-specific membrane interactions that led to cell lysis. We did attempt to determine the *in vivo* target of the inhibitors by generating resistant mutants, however this proved unsuccessful. In a previous study, Fernandez et al. used *M. smegmatis* overexpressing PknB to demonstrate that an increase in PknB levels was able to increase resistance to their PknB inhibitors.^8^ However, they were only able to see a modest 2-fold increase in resistance compared to the control strain. Our attempts to perform a similar experiment in *M. tuberculosis* proved difficult, perhaps because *M. tuberculosis* is more sensitive to overexpression of PknB than *M. smegmatis.* Even a modest increase in PknB expression was sufficient to slow growth and alter cell morphology, and the effect of overexpression on inhibitor sensitivity was not significant (K.E.A. Lougheed, unpublished data).

It is also possible that PknB may not be bound by the inhibitors when it is in a physiologically relevant configuration. In other drug discovery efforts, it has been shown that small molecule inhibitors are ineffective when their targets are involved in protein–protein interactions.^52^ Such signalling complexes may behave differently in cells than in solution perhaps because the binding site is masked by interacting proteins or because of differences in the *in vivo* protein conformation compared to the purified protein against which the inhibitors were screened.

Although *pknB* is an essential gene, very low levels of activity may be sufficient for survival, especially under the conditions we used to test our compounds. The *M. tuberculosis* kinases do display some redundancy, at least in the *in vitro* screens which have been used to investigate the substrates of the kinases. It is also possible that the cross-reactivity of the inhibitors against other kinases or the binding of alternative proteins results in a sequestering of the available inhibitor away from PknB. In fact, our results have demonstrated that the majority of the inhibitors tested are also able to inhibit PknF *in vitro*, although PknG was not as strongly inhibited. Ideally, a more specific inhibitor would need to be developed that could completely inhibit PknB *in vivo* without its effects being diluted by off-target binding.

Our work appears to have highlighted one of the difficulties associated with target-based drug discovery: namely, that screening a relatively small library of inhibitors *in vitro* regularly fails to yield compounds with potent *in vivo* activity. However, the suitability of PknB as a drug target cannot yet be refuted. Our discovery of inhibitors with good *in vitro* activity and modest *in vivo* activity suggests that further screening on a larger scale may yet yield potent inhibitors able to completely inhibit the activity of PknB *in vivo*. To take into account the possibility of redundancy among the kinases, we believe further attempts to develop *M. tuberculosis* kinase inhibitors might usefully target a broad specificity inhibitor targeting multiple kinases as suggested by Hegymegi-Barakonyi et al.^13^

## Funding

This work was supported by MRC Technology through a grant from the MRCT Development Gap Fund (A853-0058) and by the Medical Research Council (U117585867 and U117584228).

## Competing interests

None declared.

## Ethical approval

Not required.

## Figures and Tables

**Table 1:** Structures and inhibitor activity against purified PknB and *M. tuberculosis* in an Alamar blue and an intracellular assay. IC_50_ is shown as the mean of three experimental repeats, Alamar blue and intracellular MIC is representative of three or more repeats.

Inhibitor	Structure	IC_50_ PknB μM	MIC *M. tuberculosis* μM∗	MIC Intracellular μM†
1.MRT67127		0.053	32	5
2.MRT67153		0.056	32	20
3.MRT68667		0.09	64	20
4.MRT68606		0.519	16	>20
5.MRT68572		16.554	16	>20
6.MRT68634		1.499	64	>20
7.MRT67131		0.345	125	>20
8.MRT67150		0.055	32	5
9.MRT67155		0.096	64	10
10.MRT67156		0.092	16	5
11.MRT67319		6.194	32	10
12.MRT68664		0.065	64	5

* 90% inhibition fluorescence compared to untreated control.

† 99% inhibition compared to untreated control.
